# Early anthracycline cardiotoxicity in adolescents and young adults with sarcoma: a prospective echocardiographic study

**DOI:** 10.1093/eschf/xvag143

**Published:** 2026-06-19

**Authors:** Efstratios Koutroumpakis, Efthymios Triantafyllou, John Andrew Livingston, Juhee Song, Claire Viguet, Andres Hughes, Savannah V Rauschendorfer, Prince Jeyabal, Theresa A Honey, Joya Chandra, Najat C Daw, Michelle A T Hildebrandt, Jose Banchs, Susan C Gilchrist, Michael E Roth, Anita Deswal, Eugenie S Kleinerman

**Affiliations:** Department of Cardiology, Division of Internal Medicine, The University of Texas MD Anderson Cancer Center, 1515 Holcombe Blvd # 1451, Houston, TX 77030, USA; Department of Cardiology, Division of Internal Medicine, The University of Texas MD Anderson Cancer Center, 1515 Holcombe Blvd # 1451, Houston, TX 77030, USA; Department of Sarcoma Medical Oncology, Division of Cancer Medicine, The University of Texas MD Anderson Cancer Center, Houston, TX, USA; Department of Biostatistics, The University of Texas MD Anderson Cancer Center, Houston, TX, USA; Department of Cardiology, Division of Internal Medicine, The University of Texas MD Anderson Cancer Center, 1515 Holcombe Blvd # 1451, Houston, TX 77030, USA; Department of Cardiology, Division of Internal Medicine, The University of Texas MD Anderson Cancer Center, 1515 Holcombe Blvd # 1451, Houston, TX 77030, USA; Department of Health, Human Performance, and Recreation, Robbins College of Health and Human Sciences, Baylor University, Waco, TX, USA; Division of Pediatrics, The University of Texas MD Anderson Cancer Center, Houston, TX, USA; Division of Pediatrics, The University of Texas MD Anderson Cancer Center, Houston, TX, USA; Division of Pediatrics, The University of Texas MD Anderson Cancer Center, Houston, TX, USA; Division of Pediatrics, The University of Texas MD Anderson Cancer Center, Houston, TX, USA; Department of Lymphoma/Myeloma, Division of Cancer Medicine, The University of Texas MD Anderson Cancer Center, Houston, TX, USA; Division of Cardiology, University of Colorado School of Medicine, Aurora, CO, USA; Division of Cardiology, Department of Medicine, University of North Carolina at Chapel Hill, Chapel Hill, NC, USA; Division of Pediatrics, The University of Texas MD Anderson Cancer Center, Houston, TX, USA; Department of Cardiology, Division of Internal Medicine, The University of Texas MD Anderson Cancer Center, 1515 Holcombe Blvd # 1451, Houston, TX 77030, USA; Division of Pediatrics, The University of Texas MD Anderson Cancer Center, Houston, TX, USA

**Keywords:** Doxorubicin, Cardiotoxicity, Chemotherapy, Early echo findings, Sarcoma survivors

## Abstract

**Background:**

Adolescents and young adults (AYAs) with sarcomas often receive high-dose doxorubicin (Dox), but data on early cardiotoxicity in this population are limited.

**Objectives:**

To prospectively evaluate early echocardiographic changes in AYAs with sarcoma treated with high-dose Dox.

**Methods:**

AYAs (15–39 years) with sarcoma treated at a tertiary cancer centre (2018–22) were prospectively enroled. Echocardiograms were performed at baseline, 1 and 2 years after cancer therapy initiation and interpreted by a single cardiologist. The primary endpoint was a >10% absolute reduction in left ventricular ejection fraction (LVEF), an absolute LVEF <50%, or >10% decrease in LV wall thickness/dimension (LVWT/D) ratio from baseline. Secondary endpoints included longitudinal changes in cardiac structure, chamber volumes, systolic and diastolic function, and strain.

**Results:**

Of 70 patients, 56 completed at least two of three study echocardiograms (median age 22.6 [IQR, 17.6–30.5] years; 41% female, 84% white). Median cumulative Dox dose was 450 (IQR, 370–450) mg/m^2^; 75% received dexrazoxane. The primary endpoint was met by 44.4% at 1 year and 27.5% at 2 years, driven primarily by LVWT/D ratio decline (37% at 1 year, 25% at 2 years), while significant LVEF decline was observed in 11.1% and 2.5%, respectively. Significant absolute changes at 1 year included LVEF (−2.73 ± 4.3%, *P* < .001), global longitudinal strain magnitude (−1.37 ± 2.56%, *P* = .002), septal e′ (−1.75 ± 2.48 cm/s, *P* < .001), and lateral e′ (−2.78 ± 3.44 cm/s, *P* < .001), persisting at 2 years. One patient (1.8%) developed ventricular fibrillation and heart failure with reduced ejection fraction, with LVEF recovery within 1 year.

**Conclusions:**

Over one-third of AYAs with sarcoma met the primary endpoint at 1 year, with half of these abnormalities persisting at 2 years, primarily driven by LVWT/D ratio reductions. Subclinical changes in strain and diastolic function were observed, reflecting the broad cardiac impact of high-dose Dox in this population.

## Graphical Abstract

**Structured graphical abstract:**
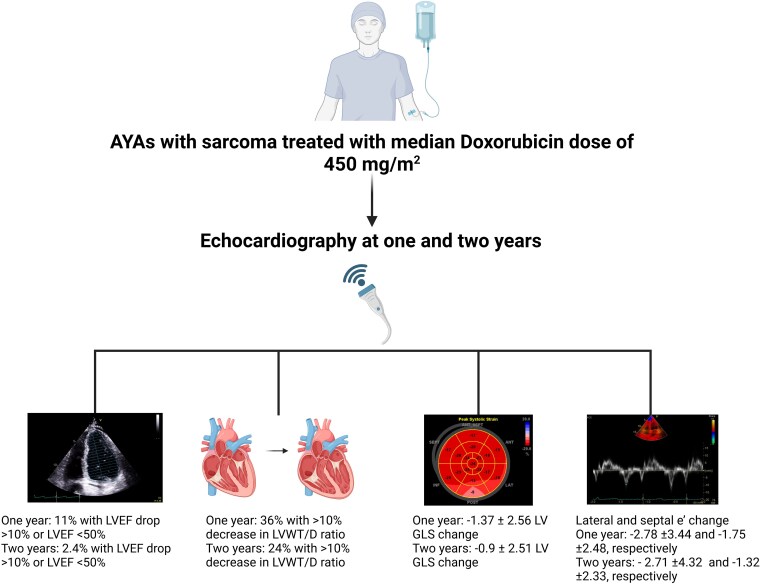
Early echocardiographic changes in AYAs with sarcoma treated with doxorubicin. Among 56 AYAs with sarcoma treated with high-dose doxorubicin, significant echocardiographic changes were observed at 1 and 2 years after treatment initiation, affecting left ventricular systolic function (expressed as drop >10% in LVEF and LVEF <50%), myocardial remodelling (expressed as a LVWT/D ratio drop >10% from baseline), diastolic function (significant drop in absolute lateral and septal e′), and myocardial deformation/global longitudinal strain (significant drop in absolute peak systolic GLS). Abbreviations: AYAs, adolescents and young adults; GLS, global longitudinal strain; LVEF, left ventricular ejection fraction; LVWT/D, left ventricular posterior wall thickness to end-diastolic diameter. Created in BioRender. Triantafyllou, E. (2026) https://BioRender.com/afhlk91

## Introduction

Cardiovascular disease has emerged as a leading cause of long-term morbidity and mortality among survivors of adolescent and young adult (AYA) cancers (ages 15–39 years at diagnosis), whose overall 5-year survival now approaches 90%.^1^ Anthracycline-based chemotherapy remains a cornerstone in the treatment of the most prevalent cancers in AYAs—including lymphomas, leukaemias, breast cancer, and sarcomas—but it can be associated with significant cardiovascular toxicity, the most concerning manifestation being anthracycline-induced cardiomyopathy and heart failure.^2^

The risk of anthracycline-induced cardiomyopathy increases with cumulative dose, particularly beyond 250 mg/m^2^ of doxorubicin (Dox)-equivalent exposure, a threshold originally established in survivors of childhood cancer.^3^ AYAs with sarcoma represent a uniquely vulnerable population, as treatment regimens often exceed 300 mg/m^2^ of Dox-equivalent dose.^4^ Additional factors—such as pre-existing cardiovascular disease, traditional cardiovascular risk factors, tobacco use, obesity, and exposure to other cardiotoxic therapies (including chest radiation) may further contribute to risk. However, pre-existing cardiovascular disease and traditional cardiovascular risk factors are relatively uncommon in AYAs, while the independent impact of obesity, tobacco use, and other cardiotoxic cancer therapies remains poorly defined in this population.^5^ Historically, the diagnosis of anthracycline-induced cardiomyopathy has relied on detecting a decline in left ventricular ejection fraction (LVEF), a change that can occur years after treatment completion, thereby limiting opportunities for early intervention and increasing the likelihood of irreversible cardiac injury.^6^

Emerging evidence suggests that subclinical echocardiographic abnormalities—such as diastolic dysfunction, reductions in the left ventricular wall thickness-to-dimension (LVWT/D) ratio, and impaired left ventricular (LV) myocardial deformation (strain)—precede overt systolic decline and may represent a critical window for early detection and cardioprotective intervention against anthracycline-induced cardiomyopathy.^5,7^ However, data defining the role of both traditional and novel imaging markers in AYAs following anthracycline exposure remain limited, particularly during the early phase after therapy completion. Most available evidence derives from studies in survivors of childhood cancer or older adults with cancer, and these studies are predominantly *retrospective* and subject to selection bias. Addressing this knowledge gap is essential to optimize surveillance strategies and inform prevention trials before irreversible myocardial injury develops. In this *prospective* study, we sought to characterize the trajectory of early cardiotoxicity and cardiac changes detected by serial echocardiographic monitoring after high-dose dox, with the goal of advancing evidence-based surveillance approaches and enabling timely interventions to prevent lifelong heart failure in AYA cancer survivors.

## Methods

### Study population

Adolescent and young adult patients aged 15–39 years with a diagnosis of sarcoma who were scheduled to receive high-dose Dox (≥300 mg/m^2^) at a single tertiary cancer centre between 2018 and 2022 were prospectively enroled. Patients were excluded if they had a history of cardiac symptoms suggestive of heart failure (e.g. dyspnoea or oedema of cardiac origin), a baseline LVEF <50% by the biplane method of disks, or LV end-diastolic or end-systolic volumes exceeding two standard deviations above the indexed normal reference values. Baseline demographic, cardiovascular risk factors, oncologic, and treatment characteristics were collected prospectively upon enrolment prior to treatment initiation. High-sensitivity troponin T (fifth generation assay; lower detection limit 6 ng/L) was measured at each study visit. The study (PA18-0462) was approved by the Institutional Review Board, and written informed consent was obtained from all participants or their legally authorized guardians as appropriate.

### Echocardiographic assessment

Transthoracic echocardiograms were performed at baseline (prior to initiation of Dox therapy) and at predefined follow-up time points at 1 and 2 years after treatment initiation. All studies were conducted by certified sonographers in accordance with the American Society of Echocardiography guidelines for performing a comprehensive transthoracic echocardiographic examination.^8^ Image interpretation was performed by a single board-certified cardiologist blinded to clinical data and in accordance with American Society of Echocardiography interpretation standards.^9^ All studies were performed using GE Vivid E95 ultrasound systems (GE Healthcare), with software versions 203–206. Global longitudinal strain (GLS) was assessed using vendor-specific 2D speckle-tracking echocardiography software, with frame rates maintained between 60 and 90 frames per second. Automated myocardial tracking was performed with manual adjustment of regions of interest as needed, and tracking quality was visually verified in all segments. No third-party or offline post-processing software was used.

Each examination included comprehensive assessment of cardiac structure and function. LV geometry was evaluated by measuring posterior wall thickness, end-diastolic and end-systolic diameter, as well as LV end-diastolic and end-systolic volumes indexed to body surface area using the biplane method of disks. LV systolic function was quantified by LVEF, and myocardial performance index (MPI) was calculated by spectral and tissue Doppler. Diastolic function was assessed from mitral inflow (E and A wave velocities), tissue Doppler lateral and septal e′ velocities, E/e′ ratio, and left atrial volume index. Right ventricular systolic function was evaluated using tricuspid annular plane systolic excursion (TAPSE) and tricuspid annular systolic velocity. LV myocardial deformation was determined by peak systolic GLS, averaged from apical four-, two-, and three-chamber views. Intracardiac filling pressures were examined using the E/e′ ratio and peak tricuspid regurgitation velocity. The LVWT/D ratio was calculated from parasternal long-axis views as the ratio of LV posterior wall thickness to LV internal diameter, both measured at end diastole.

### Endpoints

Our pre-specified primary composite endpoint was: (i) a >10-percentage-point reduction in LVEF from baseline or an absolute LVEF <50%, or (ii) a >10% decrease in the LVWT/D ratio at 1 or 2 years after Dox exposure compared with baseline. We selected LVEF because it is the conventional metric used to define cancer therapy-related cardiac dysfunction. The LVWT/D ratio was included based on paediatric data showing that it is the most common early echocardiographic abnormality after anthracycline exposure, typically emerges within the first 2 years, progresses in a predictable pattern, and is associated with subsequent development of severe cardiomyopathy.^5,10–12^ It is important to note that the prognostic significance of LVWT/D ratio has not been validated in the AYA population, and its inclusion in the primary endpoint was intended to be hypothesis-generating.

Secondary endpoints included longitudinal changes in echocardiographic indices of cardiac structure, chamber volumes, left and right ventricular systolic function, diastolic function, and myocardial deformation. Changes were assessed across three intervals: baseline to 1 year, 1–2 years, and baseline to 2 years. Echocardiographic measures were also compared with published age- and sex-specific normative values from the general population.^13,14^

### Statistical analysis

Continuous variables are presented as mean ± standard deviation or median (interquartile range [IQR]), and categorical variables as frequency (percentage). Absolute changes in echocardiographic parameters were calculated for the three intervals described above. Paired *t*-tests were used to compare changes from baseline to each follow-up time point.

To enable standardized comparisons, *Z*-scores for LVEF, LV GLS, and LV lateral and septal e′ velocities were calculated using published age- and sex-specific nomograms.^13,14^ An additional *post hoc* sensitivity analysis compared baseline characteristics between patients who were included in and excluded from the study. In an exploratory logistic regression analysis, we examined baseline demographic, clinical, and treatment characteristics as potential predictors of the primary composite endpoint at either 1 or 2 years. Results are reported as odds ratios with 95% confidence intervals. For the exploratory logistic regression analysis, baseline high-sensitivity troponin T was categorized as a three-level variable using the median of detectable values as the cut-point: undetectable, low-detectable, and above-median detectable, with the low-detectable category as the reference. *P*-value less than .05 indicated statistical significance. SAS 9.4 (SAS Institute Inc., Cary, NC) was used for data analysis.

## Results

### Cohort characteristics

**Figure 1:**
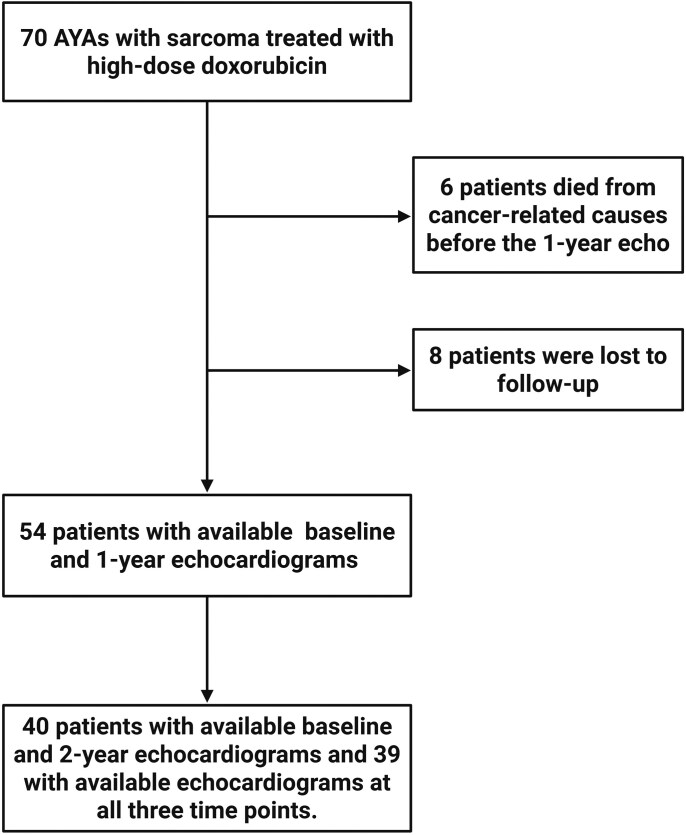
Study flow-chart. Created in BioRender. Triantafyllou, E. (2026) https://BioRender.com/tpp7ksr

A total of 70 patients were enroled, of whom 56 had at least two echocardiograms available and were included in the final analysis (six patients died due to cancer-related causes and eight were lost to follow-up before the 1-year echocardiogram; 54 had baseline and 1-year echocardiogram, 40 baseline and 2-year echocardiogram, and 39 all three echocardiograms) (*Figure 1*). Among the 56 patients, 23 (41.1%) were female, and 47 (83.9%) were White, with a median age at cancer diagnosis of 22.6 (IQR, 17.6–30.5) years and a median body mass index of 24.2 kg/m^2^ (IQR 21.6–32.5). Baseline comorbidities included dyslipidemia in three patients (5.4%), hypertension in 1 (1.8%), and type I diabetes mellitus in 1 (1.8%). Twelve patients (21.4%) reported tobacco use. Osteosarcoma was the most common malignancy (30.4%), followed by synovial sarcoma (23.2%), Ewing sarcoma (12.5%), and liposarcoma (8.9%). The median cumulative Dox dose was 450 (IQR, 370–450) mg/m^2^. Additional agents included vinca alkaloids (37.5%), nucleoside analogues or precursors (30.4%), etoposide (25%), and other agents (26.8%). Dexrazoxane was administered to 42 patients (75%) prior to each cycle of Dox, starting with cycle one. Additional demographic and clinical data are presented in *Table 1*.

**Table 1:** Baseline demographic and clinical characteristics of AYAs with sarcoma treated with high-dose doxorubicin

Covariate	*N* (%)
Age (years), median (IQR)	22.6 (17.6–30.6)
Female	23 (41.1)
Hispanic ethnicity, *n* = 54	16 (28.6)
Race	
White	47 (83.9)
Black/African American	3 (5.4)
Asian	6 (10.7)
BMI (kg/m^2^), median (IQR)	24.2 (21.6–32.5)
Smoking status	
Former	12 (21.4)
Never	44 (78.6)
Baseline CV comorbidities	5 (8.9)
HTN	1 (1.8)
HLD	3 (5.4)
DM	1 (1.8)
Obesity (BMI >30 kg/m^2^)	18 (33%)
Cancer diagnosis	
Osteosarcoma	17 (30.4)
Synovial sarcoma	13 (23.2)
Ewing sarcoma	7 (12.5)
Liposarcoma	5 (8.9)
Other	14 (25)
Disease stage, *n* = 55	
Stage 1	6 (10.9)
Stage 2	16 (29.1)
Stage 3	14 (25.5)
Stage 4	19 (34.5)
Tumor site	
Long bone	14 (25)
Other bone	8 (14.3)
Soft tissue	25 (44.6)
Thorax	6 (10.7)
Other	3 (5.4)
ECOG score	
ECOG 0	31 (55.4)
ECOG 1	18 (32.1)
ECOG 2	5 (8.9)
ECOG 3	2 (3.6)
Dexrazoxane use	
Yes	42 (75)
No	14 (25)
Systemic cancer therapies	
Doxorubicin	56 (100)
High-dose doxorubicin (>250 mg/m^2^)	53 (94.6)
Median doxorubicin dose in mg/m^2^, median (IQR)	450 (370–450)
Vinca alkaloids	21 (37.5)
Alkylating agents	56 (100%)
Ifosfamide	47 (84)
Cumulative ifosfamide dose, g/m^2^, mean (SD)	61.5 (22.7)
Etoposide	14 (25)
Nucleoside analogues and precursor	17 (30.4)
Tyrosine kinase inhibitors (TKI)	9 (16)
Other chemotherapy	15 (26.8)
Chest radiotherapy	11 (19.6%)

Abbreviations: CV, cardiovascular; DM, diabetes; HLD, hyperlipidaemia; HTN, hypertension

Note: Values are expressed as mean ± SD for normally distributed variables, median [IQR] for skewed continuous variables, and number (percentage) for categorical variables.

High sensitivity troponin T was assessed at each study visit. Troponin was detectable (≥6 ng/L) in 29 of 55 (53%), 30 of 54 (56%), and 25 of 49 (51%) patients at baseline, 1 year, and 2 years, respectively. Among patients with detectable levels, median troponin was 10.0 ng/L (IQR 8.0–13.0) at baseline, 8.5 ng/L (IQR 8.0–11.8) at 1 year, and 10.0 ng/L (IQR 7.0–16.0) at 2 years. A troponin increase was observed in 36% at 1 year and 18% at 2 years.

Excluded patients (*n* = 14) had similar baseline demographic and cardiovascular characteristics to included patients, but were more likely to have advanced cancer stage and had differences in chemotherapy regimens (Supplemental Appendix).

**Figure 2:**
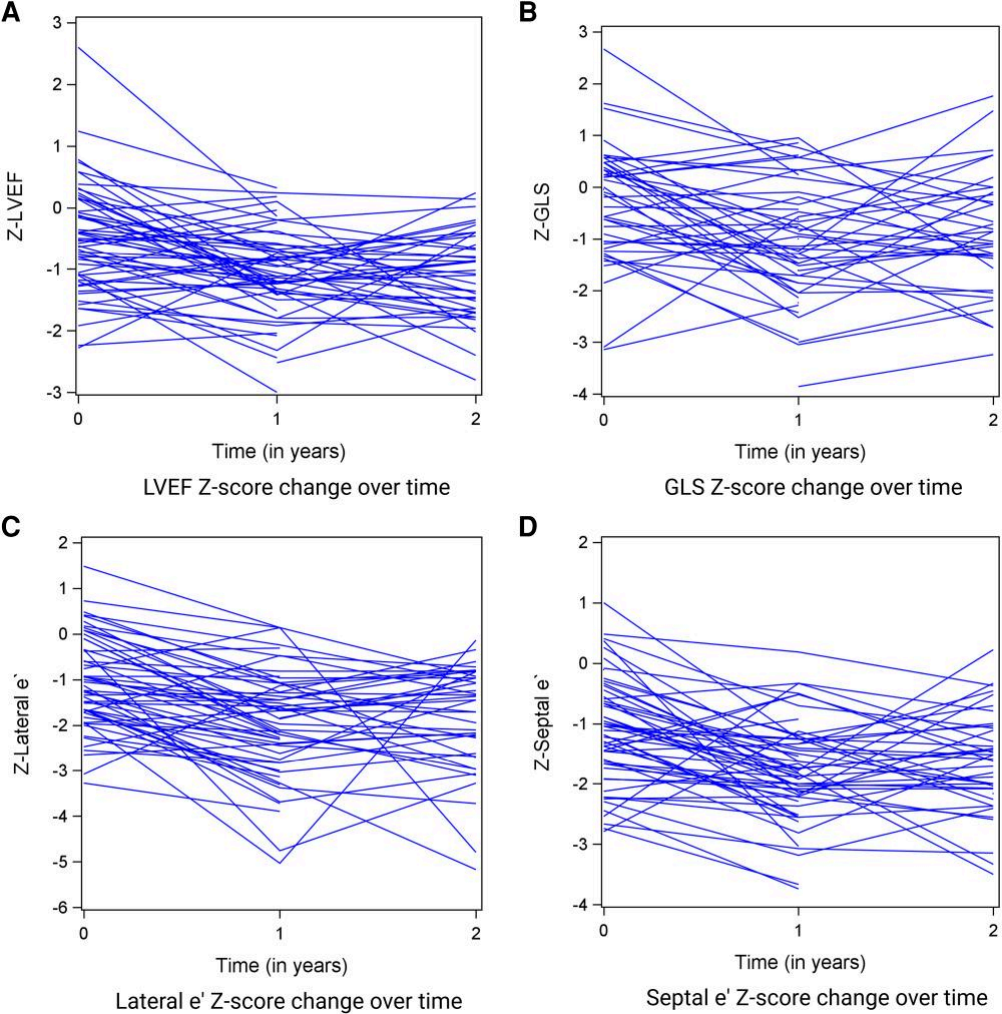
Profile plot of LVEF, GLS, lateral and septal e′ *Z*-scores over time. This figure illustrates the longitudinal changes in LVEF, GLS, lateral and septal e′ *Z*-scores for each patient at baseline, 1 and 2 years.

At baseline, among patients with both baseline and 1-year follow-up echocardiograms, the mean LVEF was 60.3 ± 4.2%, LV GLS −20.66 ± 2.4%, and lateral and septal e′ velocities 16.8 ± 3.2 cm/s and 12.3 ± 2.3 cm/s, respectively. Corresponding standardized *Z*-scores were: LVEF −0.49 ± 0.89, LV GLS −0.31 ± 1.14, LV lateral e′ −1.03 ± 1.03 and septal e′ −1.14 ± 0.90 (*Figure 2*). Additional baseline echocardiographic parameters are presented in *Table 2*.

**Table 2:** Longitudinal echocardiographic changes in AYAs with sarcoma treated with high-dose doxorubicin including all available pairs

Covariate	*N*	Baseline	1-year	*P*-value baseline to 1 year	*N*	Baseline	2-year	*P*-value baseline to 2 years
Functional measures								
LVEF (%)	53	60.32 ± 4.22	57.59 ± 3.55	**<**.**001**	40	59.98 ± 3.48	57.54 ± 3.65	.**001**
GLS magnitude (%)	41	20.66 ± 2.4	19.29 ± 2.28	.**002**	31	20.64 ± 2.13	19.74 ± 2.2	.055
Lateral e′ (cm/s)	52	16.8 ± 3.22	14.01 ± 3.42	**<**.**001**	39	16.86 ± 3.29	14.15 ± 3.47	**<**.**001**
Septal e′ (cm/s)	52	12.34 ± 2.34	10.59 ± 2.21	**<**.**001**	38	12.33 ± 2.31	11.01 ± 2.18	.**001**
MPI Ave	51	0.41 ± 0.08	0.44 ± 0.1	.**038**	38	0.41 ± 0.08	0.45 ± 0.08	.**008**
TAPSE (mm)	51	2.52 ± 0.39	2.32 ± 0.42	.**007**	38	2.44 ± 0.36	2.23 ± 0.35	.**002**
Structural measures								
LV PWT (mm)	54	8.6 (7.6–9.3)	8.1 (7.3–9)	.12	40	8.2 (7.2– 9.2)	8.1 (7.5–8.9)	.97
LV EDd (mm)	54	46.78 ± 4.56	46.83 ± 5.56	.93	40	46.15 ± 4.49	46.15 ± 4.9	1.00
LVWT/D ratio	54	0.2 ± 0.17	0.18 ± 0.03	.27	40	0.21 ± 0.2	0.18 ± 0.03	.40
EDV Index (mL/m^2^)	54	60.03 ± 13.89	56.16 ± 11.5	.**042**	40	60.53 ± 14.12	55.05 ± 11.43	.**023**
Other measures								
E/e′ ave	50	5.93 (4.95–6.45)	6.45 (5.68–7.23)	**<**.**001**	38	5.93 (4.94– 6.4)	5.95 (5.56– 7.95)	.11

Abbreviations: EDd, end-diastolic diameter; EDV, end-diastolic volume; GLS, global longitudinal strain; LV, left ventricle; LVEF, left ventricular ejection fraction; LVWT/D, left ventricle posterior wall thickness to end-diastolic diameter, MPI Ave, myocardial performance index average; PWT, posterior wall thickness; TAPSE, tricuspid annular plane systolic excursion

Note: Values are expressed as mean ± SD for normally distributed variables, and as median [IQR] for skewed continuous variables.

### Echocardiographic changes at 1 and 2 years after Dox initiation

The composite primary endpoint—defined as an absolute decrease in LVEF >10%, an LVEF <50%, or >10% decrease in the LVWT/D ratio was observed in 44.4% of patients at 1 year and 27.5% at 2 years (Central Illustration). Among patients with echocardiograms at all three time points, 38.5% met the primary endpoint at 1 year, of which 54% recovered and 46% persisted at 2 years, while 7.7% met the primary endpoint only at 2 years. Regarding the individual components, a significant LVEF decline (>10%) or LVEF <50% occurred in 6 patients (11%) at 1 year and in one patient (2.4%) at 2 years. Three patients had an LVEF below 50% at 1 year, ranging from 45% to 49%. None of the patients had a reduction of LVEF >10% to an absolute LVEF value of <50%. Of the six patients who had a >10% LVEF decline or an absolute LVEF <50% at 1 year, 2 had follow-up echocardiograms at 2 years, and both had improvement in their LVEF. Decline of >10% in the LVWT/D ratio was present in 37% of patients at 1 year and 25% at 2 years. Among patients with available echocardiograms at all three time points, a >10% decline in the LVWT/D ratio was noted in 36% at 1 year in 50% of whom it persisted at 2 years, while 5.1% developed a new decline at 2 years.

From baseline to 1 year, significant absolute reductions were observed in LVEF (−2.73 ± 4.3%, *P* < .001), LV GLS (1.37 ± 2.56%, *P* = .002), lateral e′ (−2.78 ± 3.44 cm/s, *P* < .001), and septal e′ (−1.75 ± 2.48 cm/s, *P* < .001), and the corresponding *Z*-scores declined significantly as well (*Table 3*; *Figure 2*). These abnormalities persisted at 2 years among individuals with both baseline and 2-year echocardiograms available. Additional significant changes were observed from baseline to 2 years in the tissue Doppler-derived MPI (MPI-TD average; absolute change 0.04 [IQR −0.02 to 0.1], *P* = .008), TAPSE (−0.22 ± 0.4, *P* = .002), and indexed LV end-diastolic volume (−5.48 ± 14.61, *P* = .023).

**Table 3:** Echocardiographic normative values and *Z*-scores for patients with available echocardiograms at all three time points

Covariate	Normative values	*N*	Baseline	1-year	Change	*P*-value	2-year	Change	*P*-value
EF *Z*-score^13^		38	−0.58 ± 0.73	−1.01 ± 0.56	−0.43 ± 0.75	.001	−1.07 ± 0.71	−0.49 ± 0.88	.002
Male	62 ± 5								
Female	64 ± 5								
GLS *Z*-score^13^	21.3 ± 2.1	29	−0.32 ± 1.02	−0.87 ± 1.01	−0.55 ± 1.2	.02	−0.74 ± 1.08	−0.42 ± 1.23	.08
lateral e′ *Z*-score^14^		37	−0.94 ± 1	−1.81 ± 1.17	−0.87 ± 1.21	<.001	−1.79 ± 1.06	−0.85 ± 1.31	<.001
Age 16–20	20.6 ± 3.8								
Age 21–40	19.8 ± 2.9								
septal e′ *Z*-score^14^		37	−1.12 ± 0.91	−1.63 ± 0.76	−0.51 ± 0.89	.001	−1.63 ± 0.83	−0.51 ± 0.95	.002
Age 16–20	14.9 ± 2.4								
Age 21–40	15.5 ± 2.7								

Abbreviations: EF, ejection fraction; GLS, global longitudinal strain

Normative values and corresponding *Z*-scores for LVEF, LV global longitudinal strain (GLS), and lateral and septal e′ velocities at baseline, 1 year, and 2 years after high-dose doxorubicin exposure in patients with sarcoma who had echocardiographic measurements available at all three time points

In a sensitivity analysis, comparing dexrazoxane-treated (*n* = 42) vs untreated patients (*n* = 14), no significant differences were observed in echocardiographic changes at 1 or 2 years (Supplemental Appendix). In an exploratory logistic regression analysis, no significant predictors of the primary composite endpoint at either 1 or 2 years were identified across baseline clinical and treatment variables (Supplemental Appendix).

### Cardiovascular events and mortality

During a median follow-up of 3.4 years (IQR 2.7–4.1), two patients (3.6%) developed pericardial disease with cardiac tamponade, and 1 (1.8%) cardiac arrest with ventricular fibrillation in the setting of hypokalaemia and prolonged QT interval. The incident occurred approximately 3 months after cancer treatment initiation. The patient was resuscitated with an LVEF of 35% post arrest, which improved with neurohormonal blockade to 54% seven months later. The patient expired 19 months after cancer treatment initiation due to cancer progression. Fourteen patients (25%) died due to cancer progression (median time to death for those who died: 23.2 months [IQR, 18.9–31.7]).

## Discussion

To our knowledge, this is the first prospective study to longitudinally evaluate early anthracycline-related cardiotoxicity using serial echocardiography in AYA patients with sarcoma treated with high-dose Dox. We found that: (i) more than one in three AYAs met the composite endpoint of LVEF decline and/or LVWT/D ratio at 1 year, and half of those patients had persistent abnormalities at 2 years following Dox exposure—findings primarily driven by reductions in LVWT/D ratio; (ii) development of significant LV dysfunction (LVEF < 40%) was rare during the first 2 years after cancer therapy (one patient; 1.8%); (iii) beyond LV systolic function and LVWT/D ratio, significant changes were also observed in LV deformation (strain), LV diastolic function, MPI, and right ventricular systolic function, underscoring the multidimensional cardiac impact of anthracycline cardiotoxicity; and (iv) AYAs with sarcoma exhibited lower baseline echocardiographic indices of LV function compared with published age- and sex-specific normative data from the general population, with further deterioration at 1 year and persistence through 2 years post-therapy in a subset of patients.

Anthracycline-based chemotherapy is known to cause both acute/early (during or within the first year after treatment) and chronic (years to decades) cardiotoxicity.^15^ In the largest prospective adult study including 2625 patients who underwent serial echocardiography over a median follow- up of 5.2 years, 9% developed cardiotoxicity—defined as an absolute LVEF decrease >10% to <50%—at a median of 3.5 months after treatment completion, and 1.6% developed symptomatic heart failure (NYHA class III–IV).^2^ Most events occurred within the first year after treatment completion (early-onset cardiotoxicity), with partial or full recovery in 82% after initiation of neurohormonal blockade, highlighting the benefit of early detection and intervention. The mean age of the patients in that study was 50 years; most were female (74%) with breast cancer (51%) or lymphoma (28%) treated with a cumulative Dox-equivalent dose of 360 mg/m^2^. In our AYA sarcoma cohort—markedly younger, largely free of comorbidities, with most receiving dexrazoxane yet exposed to higher cumulative Dox doses (median 450 mg/m^2^)—11% developed either a >10% decline in LVEF or an LVEF <50% at 1 year after Dox initiation—corresponding approximately to 3–6 months after treatment completion in the adult study. Notably, no patient met both LVEF criteria simultaneously, as observed in the adult cohort. One patient (1.8%) experienced ventricular fibrillation related to QTc prolongation with post-arrest reduced ejection fraction, achieving full recovery after initiation of neurohormonal therapy. Among two additional patients with reduced LVEF at 1 year who had 2-year follow-up echocardiograms, both recovered LV function. Compared with the above study and other older adult cohorts, our study is unique in evaluating AYAs with sarcoma—individuals with minimal baseline cardiovascular risk, higher anthracycline exposure, and frequent dexrazoxane use—demonstrating less prominent and mostly subclinical early declines in LVEF.

Chronic anthracycline cardiotoxicity, primarily cardiomyopathy, is well described in adults with breast cancer and lymphoma, as well as in childhood cancer survivors, and can manifest decades after treatment completion, often being largely irreversible.^16,17^ In a large cohort of 1820 adult survivors of childhood cancer (median 23 years from diagnosis; range: 10–48) exposed to anthracycline chemotherapy and chest radiotherapy, 5.8% had abnormal 3D LVEFs (<50%) with an additional 32.1% of survivors with normal 3D LVEFs having evidence of cardiac dysfunction by GLS (28%), and/or LV diastolic dysfunction (8.7%).^16,17^ The prognostic value of early echocardiographic abnormalities detected within the first 2 years after anthracycline therapy—and their relationship to the later development of chronic cardiomyopathy—remains poorly defined. Systematic identification of such early abnormalities could enable closer monitoring and timely cardioprotective interventions, potentially improving reversibility of cardiac dysfunction and mitigating the risk of irreversible heart failure, as demonstrated by Cardinale et al.^2^ and supported by our findings. To date, no comparable prospective studies have systematically evaluated early echocardiographic changes in AYAs receiving high dose Dox within the first 2 years after therapy.

In our prior *retrospective* study of 18 AYAs with osteosarcoma treated with high-dose anthracyclines, we observed a significant LVEF decline at 2 years, with 44% demonstrating a >10% reduction, along with decreases in mitral E-wave velocity and LVWT/D ratio.^5^ Building on those observations, our current *prospective* study confirmed a statistically significant but more modest decline in LVEF at 1 year and 2 years. In the current study, the proportion of patients meeting formal criteria for cardiotoxicity (LVEF drop >10% or <50%) was smaller—11% at 1 year and 2.4% at 2 years, with no patient meeting both criteria, whereas a significant decline in LVWT/D ratio was evident in 36% and 24%, respectively. The less pronounced LVEF decline likely reflects differences in patient selection and treatment era, including a lower cumulative mean Dox dose (399 vs 436 mg/m^2^) and more frequent use of dexrazoxane (75% vs 33%).

A substantial proportion of our patients exhibited subclinical structural and functional echocardiographic abnormalities within the first 2 years after Dox, underscoring persistent vulnerability even in the modern oncology era and despite wide use of dexrazoxane. Beyond changes in LVEF, we observed significant impairments in LV strain, diastolic function, MPI, and right ventricular systolic function—findings consistent with adult studies demonstrating that abnormalities in diastolic function and myocardial deformation often precede overt declines in LVEF in anthracycline-induced cardiomyopathy.^18,19^ We also noted a lower prevalence of echocardiographic abnormalities at 2 years compared with 1 year, suggesting that not all changes are progressive. Long-term follow-up will be critical to determine whether patients with early echocardiographic findings experience subsequent recovery or progressive dysfunction. Notably, we also observed a significant decrease in indexed LV end-diastolic volume at 2 years compared with baseline. We suspect that this may reflect variations in loading conditions or afterload at the time of each echocardiogram, as well as potential technical limitations in volume tracing, rather than a true reduction in LV volumes. However, this finding warrants further evaluation in larger studies.

Another important observation is that, even before chemotherapy initiation, AYAs with sarcoma demonstrated numerically lower indices of LV systolic and diastolic function compared with age- and sex-specific normative values, suggesting an intrinsic cardiac vulnerability that may be related to underlying disease biology, inflammation, or heightened physiologic stress.^20^ These abnormalities worsened after anthracycline exposure, emphasizing that conventional definitions of cardiotoxicity—relying primarily on absolute LVEF thresholds—may lack sensitivity for detecting early myocardial injury in this population. Incorporating age- and sex-specific reference values and evaluating relative changes from individualized baselines may improve early detection and enable timely cardioprotective interventions in AYAs, although larger studies are needed to evaluate the progression of these early changes to clinical heart failure.

Dexrazoxane was administered in three-quarters of patients and may have contributed to the relatively low rate of overt LVEF decline observed. Nevertheless, subclinical echocardiographic abnormalities were still observed in over one-third of the cohort, and in our sensitivity analysis, dexrazoxane use did not correlate with preserved LV function at 1 or 2 years compared with those who did not receive dexrazoxane. These findings should be interpreted with caution, however, as our study was not designed or powered to evaluate dexrazoxane efficacy, and the non-randomized assignment likely introduced selection bias. Larger, dedicated studies with longer follow-up, such as Chow et al., have shown that dexrazoxane mitigates overt LVEF decline, while it may not fully prevent subclinical myocardial injury or structural remodelling.^21^ Our data provide only exploratory, hypothesis-generating evidence that supports this notion, and further investigation in prospective trials is warranted.

In an exploratory logistic regression analysis, no baseline clinical or treatment variables significantly predicted the primary composite endpoint, likely reflecting the modest sample size and the fact that this study was neither designed nor powered to identify predictors of cardiotoxicity. Larger, adequately powered studies in AYA cohorts are warranted to identify risk factors for anthracycline-induced cardiotoxicity in this population.

Currently, there is no clear evidence-based surveillance guidelines specifically addressing early cardiotoxicity detection in AYAs treated with anthracyclines. In practice, LVEF remains the primary imaging metric, but it may lack sensitivity for early myocardial injury.^22^ Our findings suggest that routine incorporation of LV strain, diastolic function, and LVWT/D ratio into longitudinal surveillance may enhance early detection and guide preventive care in this high-risk population. Long-term studies will be needed, however, to establish whether early LVWT/D ratio changes in AYAs carry the same prognostic weight as observed in childhood cancer survivors.

### Limitations

Our study has several limitations. It was conducted at a single tertiary cancer centre, which may limit generalizability and introduce selection bias. Participants likely had greater access to specialized care, potentially mitigating cardiotoxicity rates. The modest sample size may have limited statistical power to detect clinically meaningful differences. Additionally, the relatively short follow-up limits assessment of progression from early changes to overt heart failure. Left atrial strain, an emerging marker of subclinical cardiac dysfunction in the setting of anthracycline-induced cardiotoxicity, was not assessed, as its role was not widely accepted at the time of protocol development (2018). The 14 patients excluded due to insufficient echocardiographic follow-up had more advanced cancer stage and differences in chemotherapy regimens at baseline, likely reflecting survivorship bias, which may have led to underestimation of the incidence and severity of cardiotoxicity in our cohort. Finally, our exploratory analyses examining predictors of echocardiographic abnormalities were limited by sample size and should be interpreted cautiously, as the study was neither designed nor powered to identify predictors of cardiotoxicity. Larger, multicentre studies with extended follow-up are warranted to validate these findings and to determine the long-term clinical implications of early abnormalities. Despite its limitations, our study is the first to evaluate early echocardiographic abnormalities in AYAs treated with high-dose Dox, providing valuable insight into this understudied population.

## Conclusion

In conclusion, this prospective study found that over one-third of AYAs met the composite endpoint of significant decline in LVEF and/or LVWT/D ratio at 1 year, with half of these patients showing persistent abnormalities at 2 years—primarily driven by reductions in LVWT/D ratio. Significant LV dysfunction (LVEF < 40%) was rare. Beyond LV systolic function and LVWT/D ratio, significant changes were observed in LV deformation (strain), LV diastolic function, MPI, and right ventricular systolic function, highlighting the multidimensional impact of anthracycline cardiotoxicity. These early subclinical echocardiographic abnormalities may identify patients at higher risk for clinical anthracycline-induced heart failure and provide a foundation for future long-term studies aimed at refining surveillance protocols and improving early detection and prevention in this vulnerable population.

## Supplementary Material

xvag143_Supplementary_Data

## Declarations

### Data Availability

Consistent with the NIH Final Policy for Data Management and Sharing (NOT-OD-21-013), anonymized and de-identified data underlying this study will be deposited in the NIH-supported generalist repository Figshare and made openly available at http://doi.org/10.6084/m9.figshare.30569843 at or before the time of publication.

### Pre-registered Clinical Trial Number

None supplied.

## Public access policy compliance

This manuscript is the result of funding, in whole or in part, by the National Institutes of Health (NIH). It is subject to the NIH Public Access Policy in accordance with NOT-OD-25-049, *Supplemental Guidance to the 2024 NIH Public Access Policy: Government Use License and Rights*. Through acceptance of this federal funding, NIH has been granted a government use license and the right to make this manuscript publicly available in PubMed Central upon the Official Date of Publication, as defined by NIH.

## Reporting guideline compliance statement

In accordance with EQUATOR Network (Enhancing the QUAlity and Transparency Of health Research) guidance, we have utilized the STROBE (Strengthening the Reporting of Observational Studies in Epidemiology) checklist; the completed STROBE checklist is attached as Supplemental Table in the Supplemental Appendix.
