# Supplementary material for: Early anthracycline cardiotoxicity in adolescents and young adults with sarcoma: a prospective echocardiographic study
Source: ESC Heart Fail. 2026 Jun 19;13(3):xvag143. doi: 10.1093/eschf/xvag143 (PMC13282902; doi:10.1093/eschf/xvag143)
Supplement: xvag143_Supplementary_Data [file xvag143_supplementary_data.docx]

**Early Anthracycline Cardiotoxicity in Adolescents and Young Adults with Sarcoma: A Prospective Echocardiographic Study**

**Supplemental Material** **Content**

1. Supplemental Table 1: Longitudinal Subacute Echocardiographic Changes in Adolescents and Young Adults with Sarcoma Treated with High-Dose Doxorubicin (Extended). **Page 2**
2. Supplemental Table 2: Baseline Z scores for EF, GLS, Lateral e’ and Septal e’ and age/sex specific normal values. **Page 5**
3. Supplemental Table 3: Comparison between baseline and 1-year follow-up Z-scores including patients with echocardiograms at both time points. **Page 6**
4. Supplemental Table 4: Comparison between baseline and 2-year follow-up Z-scores including patients with echocardiograms at both time points. **Page 7**
5. Supplemental Table 5: Dexrazoxane vs no-dexrazoxane patient characteristics. **Page 8**
6. Supplemental Table 6: Comparison of excluded vs included patient characteristics. **Page 11**
7. Supplemental Table 7: Univariate logistic regression analysis for predictors of the primary composite endpoint at any timepoint. **Page 14**
8. Supplemental Table 8: STROBE Statement—checklist of items that should be included in reports of observational studies. **Page 16**

**Supplemental Table 1: Longitudinal Subacute Echocardiographic Changes in Adolescents and Young Adults with Sarcoma Treated with High-Dose Doxorubicin (Extended)**

| Covariate | N | Baseline | 1yr | N | Baseline | 2yr | p-value baseline to 1 year | p-value baseline to  2 years | % of patients with significant change |
| --- | --- | --- | --- | --- | --- | --- | --- | --- | --- |
| Functional measures |  |  |  |  |  |  |  |  |  |
| LVEF (%) | 53 | 60.32±4.22 | 57.59±3.55 | 40 | 59.98±3.48 | 57.54±3.65 | **<.001** | **0.001** | Y1: 3(5.7%),  Y2: 1(2.5%)0 |
| GLS magnitude (%) | 41 | 20.66±2.4 | 19.29±2.28 | 31 | 20.64±2.13 | 19.74±2.2 | **0.002** | 0.055 | Y1: 11(26.8%),  Y2: 6(19.4%) |
| Lateral e’ (cm/s) | 52 | 16.8±3.22 | 14.01±3.42 | 39 | 16.86±3.29 | 14.15±3.47 | **<.001** | **<.001** | Y1: 23(44.2%)  Y2: 14(35.9%) |
| Septal e’ (cm/s) | 52 | 12.34±2.34 | 10.59±2.21 | 38 | 12.33±2.31 | 11.01±2.18 | **<.001** | **0.001** | Y1: 18(34.6%)  Y2: 12(31.6%) |
| MPI SD | 53 | 0.36±0.13 | 0.36±0.12 | 39 | 0.37±0.13 | 0.34±0.14 | 0.82 | 0.35 |  |
| Lateral MPI TD tissue | 51 | 0.4(0.34− 0.47) | 0.43(0.33− 0.48) | 38 | 0.4(0.33−0.47) | 0.44(0.39− 0.52) | 0.22 | **0.03** |  |
| Septal MPI TD tissue | 52 | 0.42±0.09 | 0.47±0.12 | 38 | 0.42±0.1 | 0.46±0.11 | **0.02** | 0.07 |  |
| MPI Ave | 51 | 0.41±0.08 | 0.44±0.1 | 38 | 0.41±0.08 | 0.45±0.08 | **0.038** | **0.008** |  |
| TAPSE (mm) | 51 | 2.52±0.39 | 2.32±0.42 | 38 | 2.44±0.36 | 2.23±0.35 | **0.007** | **0.002** |  |
| Structural measures |  |  |  |  |  |  |  |  |  |
| LV PWT (mm) | 54 | 8.6(7.6−9.3) | 8.1(7.3 − 9) | 40 | 8.2 (7.2− 9.2) | 8.1 (7.5− 8.9) | 0.12 | 0.97 |  |
| LV EDd (mm) | 54 | 46.78±4.56 | 46.83±5.56 | 40 | 46.15±4.49 | 46.15±4.9 | 0.93 | 1.00 |  |
| LVWT/D ratio | 54 | 0.2±0.167 | 0.177±0.03 | 40 | 0.21±0.2 | 0.18±0.03 | 0.27 | 0.40 |  |
| Index EDV (mL/m²) | 54 | 60.03±13.89 | 56.16±11.5 | 40 | 60.53±14.12 | 55.05±11.43 | 0.04 | **0.02** |  |
| LAVI (mL/m²) | 51 | 19.6(16.2 − 24.8) | 19.8(16 − 24.3) | 35 | 18.5(15.8− 24) | 19.9(17.1− 24.1) | 0.75 | 0.31 |  |
| Other measures |  |  |  |  |  |  |  |  |  |
| E/A | 53 | 1.6(1.3− 1.87) | 1.48(1.2−1.94) | 39 | 1.56(1.3−1.89) | 1.69(1.4− 1.98) | 0.38 | 0.74 |  |
| E/e’ave | 50 | 5.93(4.95−6.45) | 6.45(5.68−7.23) | 38 | 5.93 (4.94− 6.4) | 5.95 (5.56− 7.95) | **<.001** | 0.11 |  |

Abbreviations: BMI - Body Mass Index, BP - Blood Pressure, BSA - Body Surface Area,  EDd-End-Diastolic Diameter, EDV - End-Diastolic Volume, GLS-Global Longitudinal Strain, LAVI - Left Atrial Volume Index, LV -Left ventricle, LVEF - Left Ventricle Ejection Fraction, LVWT/D- Left ventricle posterior wall thickness to end-diastolic diameter, MPI Ave - Myocardial Performance Index Average, PWT-Posterior Wall Thickness, TAPSE-Tricuspid Annular Plane Systolic Excursion, TD - Tissue Doppler

Note: Values are expressed as mean ± SD for normally distributed variables, and as median [IQR] for skewed continuous variables

**Supplemental Table 2: Baseline Z scores for EF, GLS, Lateral e’ and Septal e’ and age/sex specific normal values**

| Covariate | Normal values | N | Baseline |
| --- | --- | --- | --- |
| EF Z-score |  | 55 | -0.51±0.88 |
| Male | 62 ± 5 |  |  |
| Female | 64 ± 5 |  |  |
| GLS Z-score | -21.3 ± 2.1 | 44 | -0.30±1.12 |
| lateral e` Z-score |  | 54 | -1.06±1.02 |
| 16-20 | 20.6 ± 3.8 |  |  |
| 21-40 | 19.8 ± 2.9 |  |  |
| septal e` Z-score |  | 54 | -1.13±0.90 |
| 16-20 | 14.9 ± 2.4 |  |  |
| 21-40 | 15.5 ± 2.7 |  |  |

  Abbreviations: EF - Ejection Fraction, GLS - Global Longitudinal Strain

**Supplemental Table 3: Comparison between baseline and 1-year follow-up Z-scores including patients with echocardiograms at both time points**

| Covariate | N | Baseline | 1yr | Change | P-value |
| --- | --- | --- | --- | --- | --- |
| EF Z-score | 53 | -0.49±0.89 | -1.04±0.71 | -0.55±0.86 | <.001 |
| GLS Z-score | 41 | -0.31±1.14 | -0.96±1.09 | -0.65±1.22 | 0.002 |
| lateral e` Z-score | 52 | -1.03±1.01 | -1.94±1.17 | -0.91±1.08 | <.001 |
| septal e` Z-score | 52 | -1.14±0.9 | -1.8±0.84 | -0.66±0.96 | <.001 |

  Abbreviations: EF - Ejection Fraction, GLS - Global Longitudinal Strain

**Supplemental Table 4: Comparison between baseline and 2-year follow-up Z-scores including patients with echocardiograms at both time points**

| Covariate | N | Baseline | 2yr | Change | P-value |
| --- | --- | --- | --- | --- | --- |
| EF Z-score | 40 | -0.6±0.72 | -1.08±0.7 | -0.49±0.88 | 0.001 |
| GLS Z-score | 31 | -0.32±1.01 | -0.74±1.05 | -0.43±1.19 | 0.06 |
| lateral e` Z-score | 39 | -0.99±1.01 | -1.89±1.16 | -0.89±1.38 | <.001 |
| septal e` Z-score | 38 | -1.13±0.9 | -1.66±0.83 | -0.52±0.94 | 0.002 |

  Abbreviations: EF - Ejection Fraction, GLS - Global Longitudinal Strain

**Supplemental Table 5: Dexrazoxane vs no-dexrazoxane patient characteristics**

| Covariate | Dexrazoxane (n=42) | No-dexrazoxane (n=14) | P-value |
| --- | --- | --- | --- |
|  | Median (IQR) or N (%) | |  |
| Age | 21.2(17− 27.83) | 30.6(25.1− 32.9) | **0.01** |
| Female | 17 (40.5) | 6 (42.9) | 0.88 |
| Hispanic ethnicity | 12(28.6) | 4(30.8) | 1.00 |
| Race |  |  |  |
| White | 35(83.3) | 12(85.7) | 1.00 |
| Black/African American | 2(4.8) | 1(7.1) |  |
| Asian | 5(11.9) | 1(7.1) | 0.843 |
| BMI (kg/m²) | 24.9(21.8− 34.3) | 23.8(20.4− 30.1) | 0.59 |
| Smoker |  |  |  |
| Former | 10(23.8) | 2(14.3) |  |
| Never | 32(76.2) | 12(85.7) | 0.71 |
| Baseline CV Comorbidities |  |  |  |
| HTN | 1(2.4) | 0 | 1.00 |
| HLD | 2(4.8) | 1(7.1) |  |
| DM | 1(2.4) | 0 |  |
| Diagnosis |  |  |  |
| Ewing | 6(14.3) | 1(7.1) | 0.001 |
| Synovial sarcoma | 5(11.9) | 8(57.1) |  |
| Osteosarcoma | 17(40.5) | 0 |  |
| Liposarcoma | 3(7.1) | 2(14.3) |  |
| Other | 11(26.2) | 3(21.4) |  |
| Stage |  |  |  |
| Stage 1 | 2(7.3) | 3(21.4) | 0.17 |
| Stage 2 | 12(29.3) | 4(28.6) |  |
| Stage 3 | 13(31.7) | 1(7.1) |  |
| Stage 4 | 13(31.7) | 6(42.9) | 0.1 |
| Tumor Site |  |  |  |
| Long bone | 14(33.3) | 0 | 0.009 |
| Other bone | 7(16.7) | 1(7.1) |  |
| Soft tissue | 17(40.5) | 8(57.1) |  |
| Respiratory | 2(4.8) | 4(28.6) |  |
| Other | 2(4.8) | 1(7.1) |  |
| Ecog Score |  |  |  |
| Ecog 0 | 21(50) | 10(71.4) | 0.29 |
| Ecog 1 | 15(35.7) | 3(21.4) |  |
| Ecog 2 | 5(11.9) | 0 |  |
| Ecog 3 | 1(2.4) | 1(7.1) |  |
| Systemic Cancer Therapies |  |  |  |
| Doxorubicin | 42 (100) | 14(100) | - |
| High Dose Doxorubicin (>250) mg/m^2^) | 39(92.9) | 14(100) | 0.57 |
| Doxorubicin dose (mg/m^2^) | 442(366− 450) | 450(434− 450) | 0.14 |
| Vinca Alkaloids | 20(47.6) | 1(7.1) | **0.007** |
| Etoposide | 13(31) | 1(7.1) | 0.15 |
| Nucleoside analogs and precursor | 17(40.5) | 0 | **0.00** |
| Other Chemotherapy | 14(33.3) | 1(7.1) | 0.08 |

 Abbreviations: BMI - Body Mass Index, CV - Cardiovascular, DM - Diabetes, HLD - Hyperlipidemia, HTN - Hypertension

**Supplemental Table 6: Comparison of excluded vs included patient characteristics**

| Covariate | Excluded (n=14) | Included (n=56) | P-value |
| --- | --- | --- | --- |
|  | Median (IQR) or N (%) | |  |
| Age | 28.3 (8.0) | 24.1 (6.9) | 0.09 |
| Female | 7 (50%) | 23 (41%) | 0.76 |
| Hispanic ethnicity | 7 (54%) | 16 (30%) | 0.19 |
| Smoker |  |  |  |
| Former | 1 (7.1%) | 12 (21%) |  |
| Never | 13 (93%) | 44 (79%) | 0.4 |
| Baseline CV Comorbidities |  |  |  |
| HTN | 1 (8.3%) | 1 (1.8%) | 0.6 |
| HLD | 1 (8.3%) | 3 (5.4%) |  |
| DM | 0 (0%) | 1 (1.8%) |  |
| Diagnosis |  |  |  |
| Ewing | 2 (14%) | 7 (13%) | 0.48 |
| Synovial sarcoma | 5 (36%) | 13 (23%) |  |
| Osteosarcoma | 2 (14%) | 17 (30%) |  |
| Liposarcoma | 0 (0%) | 5 (8.9%) |  |
| Other | 5 (36%) | 14 (25%) |  |
| Stage |  |  |  |
| Stage 1 | 0 (0%) | 6 (11%) |  |
| Stage 2 | 1 (9.1%) | 16 (29%) |  |
| Stage 3 | 2 (18%) | 14 (25%) |  |
| Stage 4 | 7 (64%) | 19 (35%) | **0.044** |
| Ecog Score |  |  |  |
| Ecog 0 | 11 (79%) | 31 (55%) | 0.05 |
| Ecog 1 | 0 (0%) | 18 (32%) |  |
| Ecog 2 | 1 (7.1%) | 5 (8.9%) |  |
| Ecog 3 | 2 (14%) | 2 (3.6%) |  |
| Systemic Cancer Therapies |  |  |  |
| Vinca Alkaloids | 6 (43%) | 21 (38%) | 0.95 |
| Alkylating Agents | 12 (86%) | 56 (100%) | **0.049** |
| Etoposide | 4 (29%) | 14 (25%) | >0.99 |
| Nucleoside analogs and precursor | 0 (0%) | 17 (30%) | **0.043** |
| Other Chemotherapy | 7 (50%) | 15 (27%) | 0.18 |
| Dexrazoxane | 9 (64%) | 42 (75%) | 0.64 |

 Abbreviations: BMI - Body Mass Index, CV - Cardiovascular, DM - Diabetes, HLD - Hyperlipidemia, HTN - Hypertension

**Supplemental Table 7: Univariate logistic regression analysis for predictors of the primary composite endpoint at any timepoint**

| Covariate (n=48) | OR | 95% CI | p-value |
| --- | --- | --- | --- |
| Age | 1.00 | 0.92 - 1.09 | 0.92 |
| Female | 0.79 | 0.25 - 2.53 | 0.69 |
| BMI, kg/m² | 0.99 | 0.95 - 1.04 | 0.84 |
| Former smoker | 0.82 | 0.21 - 3.18 | 0.77 |
| Any baseline CV comorbidity | 1.46 | 0.12 - 17.32 | 0.76 |
| Obesity | 1.50 | 0.41 - 5.45 | 0.54 |
| Systemic Cancer Therapies |  |  |  |
| Vinca alkaloids | 2.60 | 0.74 - 9.12 | 0.14 |
| Ifosfamide | 1.50 | 0.33 - 6.88 | 0.60 |
| Etoposide | 1.20 | 0.33 - 4.41 | 0.78 |
| Nucleoside analogs | 1.11 | 0.32 - 3.83 | 0.87 |
| Other chemotherapy | 2.22 | 0.58 - 8.49 | 0.24 |
| Dexrazoxane | 2.48 | 0.65 - 9.40 | 0.18 |
| Doxorubicin dose, mg/m² | 1.00 | 0.99 - 1.00 | 0.47 |
| Chest radiotherapy | 0.39 | 0.09 - 1.62 | 0.19 |
| Troponin, baseline |  |  |  |
| Low-detectable (6-10) | 1.00 |  |  |
| Above-median detectable (>10) | 2.00 | 0.28 - 14.20 | 0.49 |
| Undetectable | 0.43 | 0.10 - 1.79 | 0.24 |
| Delta troponin, baseline to 1 year | 1.00 | 0.85 - 1.19 | 0.97 |
| Delta troponin, baseline to 2 years | 0.93 | 0.65 - 1.31 | 0.67 |

 Abbreviations: BMI - Body Mass Index, CV - Cardiovascular, DM - Diabetes, HLD - Hyperlipidemia, HTN - Hypertension

**Supplemental Table 8: STROBE Statement—checklist of items that should be included in reports of observational studies**

|  | Item No. | Recommendation | Page  No. |
| --- | --- | --- | --- |
| **Title and abstract** | 1 | (*a*) Indicate the study’s design with a commonly used term in the title or the abstract | 1 |
|  |  | (*b*) Provide in the abstract an informative and balanced summary of what was done and what was found | 4 |
| Introduction | | | |
| Background/rationale | 2 | Explain the scientific background and rationale for the investigation being reported | 6 |
| Objectives | 3 | State specific objectives, including any prespecified hypotheses | 7 |
| Methods | | | |
| Study design | 4 | Present key elements of study design early in the paper | 8 |
| Setting | 5 | Describe the setting, locations, and relevant dates, including periods of recruitment, exposure, follow-up, and data collection | 8-9 |
| Participants | 6 | (*a*) *Cohort study*—Give the eligibility criteria, and the sources and methods of selection of participants. Describe methods of follow-up  *Case-control study*—Give the eligibility criteria, and the sources and methods of case ascertainment and control selection. Give the rationale for the choice of cases and controls  *Cross-sectional study*—Give the eligibility criteria, and the sources and methods of selection of participants | 8-9 |
|  |  | (*b*) *Cohort study*—For matched studies, give matching criteria and number of exposed and unexposed  *Case-control study*—For matched studies, give matching criteria and the number of controls per case |  |
| Variables | 7 | Clearly define all outcomes, exposures, predictors, potential confounders, and effect modifiers. Give diagnostic criteria, if applicable | 8-9 |
| Data sources/ measurement | 8* | For each variable of interest, give sources of data and details of methods of assessment (measurement). Describe comparability of assessment methods if there is more than one group | *8-9* |
| Bias | 9 | Describe any efforts to address potential sources of bias | 8-10 |
| Study size | 10 | Explain how the study size was arrived at | 8 |

| Quantitative variables | 11 | Explain how quantitative variables were handled in the analyses. If applicable, describe which groupings were chosen and why |  | 10 |
| --- | --- | --- | --- | --- |
| Statistical methods | 12 | (*a*) Describe all statistical methods, including those used to control for confounding |  | 10 |
|  |  | (*b*) Describe any methods used to examine subgroups and interactions |  | 10 |
|  |  | (*c*) Explain how missing data were addressed |  | 10 |
|  |  | (*d*) *Cohort study*—If applicable, explain how loss to follow-up was addressed  *Case-control study*—If applicable, explain how matching of cases and controls was addressed  *Cross-sectional study*—If applicable, describe analytical methods taking account of sampling strategy |  | 8-10 |
|  |  | (*e*) Describe any sensitivity analyses |  | 10 |
| Results | | | | |
| Participants | 13* | (a) Report numbers of individuals at each stage of study—eg numbers potentially eligible, examined for eligibility, confirmed eligible, included in the study, completing follow-up, and analysed |  | 11 |
|  |  | (b) Give reasons for non-participation at each stage |  | 11 |
|  |  | (c) Consider use of a flow diagram |  | Fig 1 |
| Descriptive data | 14* | (a) Give characteristics of study participants (eg demographic, clinical, social) and information on exposures and potential confounders |  | 11 |
|  |  | (b) Indicate number of participants with missing data for each variable of interest |  | 11 |
|  |  | (c) *Cohort study*—Summarise follow-up time (eg, average and total amount) |  | 13 |
| Outcome data | 15* | *Cohort study*—Report numbers of outcome events or summary measures over time |  | *11-13* |
|  |  | *Case-control study—*Report numbers in each exposure category, or summary measures of exposure |  |  |
|  |  | *Cross-sectional study—*Report numbers of outcome events or summary measures |  |  |
| Main results | 16 | (*a*) Give unadjusted estimates and, if applicable, confounder-adjusted estimates and their precision (eg, 95% confidence interval). Make clear which confounders were adjusted for and why they were included |  | 11-13 |
|  |  | (*b*) Report category boundaries when continuous variables were categorized |  |  |
|  |  | (*c*) If relevant, consider translating estimates of relative risk into absolute risk for a meaningful time period |  |  |

| Other analyses | 17 | Report other analyses done—eg analyses of subgroups and interactions, and sensitivity analyses |  | 11-13 |
| --- | --- | --- | --- | --- |
| Discussion | | | | |
| Key results | 18 | Summarise key results with reference to study objectives |  | 14 |
| Limitations | 19 | Discuss limitations of the study, taking into account sources of potential bias or imprecision. Discuss both direction and magnitude of any potential bias |  | 18 |
| Interpretation | 20 | Give a cautious overall interpretation of results considering objectives, limitations, multiplicity of analyses, results from similar studies, and other relevant evidence |  | 14-17 |
| Generalisability | 21 | Discuss the generalisability (external validity) of the study results |  | 14-18 |
| Other information | |  | | |
| Funding | 22 | Give the source of funding and the role of the funders for the present study and, if applicable, for the original study on which the present article is based |  | 2 |

*Give information separately for cases and controls in case-control studies and, if applicable, for exposed and unexposed groups in cohort and cross-sectional studies.

**Note:** An Explanation and Elaboration article discusses each checklist item and gives methodological background and published examples of transparent reporting. The STROBE checklist is best used in conjunction with this article (freely available on the Web sites of PLoS Medicine at http://www.plosmedicine.org/, Annals of Internal Medicine at http://www.annals.org/, and Epidemiology at http://www.epidem.com/). Information on the STROBE Initiative is available at www.strobe-statement.org.
